# Predicting delinquent behavior in young adults with a childhood diagnosis of ADHD: results from the Cologne Adaptive Multimodal Treatment (CAMT) Study

**DOI:** 10.1007/s00787-020-01698-y

**Published:** 2020-12-04

**Authors:** Dieter Breuer, Elena von Wirth, Janet Mandler, Stephanie Schürmann, Manfred Döpfner

**Affiliations:** 1 School for Child and Adolescent Cognitive Behavior Therapy (AKiP), University of Cologne, Faculty of Medicine and University Hospital Cologne, Pohligstr. 9, 50969 Cologne, Germany; 2 Department of Child and Adolescent Psychiatry, Psychosomatics and Psychotherapy, University of Cologne, Faculty of Medicine and University Hospital Cologne, Robert-Koch-Str. 10, 50931 Cologne, Germany

**Keywords:** Attention-deficit, Hyperactivity disorder, Longitudinal study, Delinquent behavior, Prediction

## Abstract

The aim of this study was to investigate which factors predict lifetime reports of delinquent behavior in young adults who had received adaptive multimodal treatment of attention-deficit/hyperactivity disorder (ADHD) starting at ages 6–10 years. Participants were reassessed 13–24 years (*M* = 17.6, SD = 1.8) after they had received individualized ADHD treatment in the Cologne Adaptive Multimodal Treatment Study (CAMT). Their behavior was classified as non-delinquent (*n* = 34) or delinquent (*n* = 25) based on self-reports regarding the number of police contacts, offenses, and convictions at follow-up. Childhood variables assessed at post-intervention (e.g., externalizing child behavior problems, intelligence, and parenting behavior) that were significantly associated with group membership were entered as possible predictors of delinquency in a Chi-squared automatic interaction detector (CHAID) analysis. Delinquent behavior during adolescence and adulthood was best predicted by (a) meeting the symptom count diagnostic criteria for conduct disorder (CD) according to parent ratings, in combination with a nonverbal intelligence of IQ ≤ 106 at post-intervention, and (b) delinquent behavior problems (teacher rating) at post-intervention. The predictor variables specified in the CHAID analysis classified 81% of the participants correctly. The results support the hypothesis that a childhood diagnosis of ADHD is only predictive of delinquent behavior if it is accompanied by early conduct behavior problems. Low nonverbal intelligence was found to be an additional risk factor. These findings underline the importance of providing behavioral interventions that focus on externalizing behavior problems to children with ADHD and comorbid conduct problems.

## Introduction

Attention-deficit/hyperactivity disorder (ADHD) is one of the most common reasons for referral for treatment [1]. It has a prevalence rate of approximately 4% and a male-to-female ratio of about 2:1 [2, 3]. The high referral rate may, in part, be due to the fact that children with ADHD are at risk of developing oppositional, aggressive, and rule-breaking behavior problems [4]. A recent meta-analysis [5] found that ADHD is longitudinally associated with increased rates of oppositional defiant disorder (ODD; odds ratio [OR] 7.0), conduct disorder (CD; OR 5.4), and antisocial personality disorder (OR 3.2). Moreover, ADHD is associated with a higher risk of illicit drug use (OR 2.2) and of being arrested (OR 2.4). Similarly, another recent meta-analysis [6] showed that children with ADHD were more likely to be arrested (relative risk [RR] 2.2), convicted (RR 3.3) or incarcerated (RR 2.9) during adolescence or adulthood. For example, Barkley et al. [7] followed a sample of hyperactive children and a community control (CC) group into young adulthood and found that the hyperactive group had committed more antisocial acts, including theft, disorderly conduct, assault with fists, carrying a concealed weapon, and illegal drug possession. They had also been arrested more often than the control group (felony arrest: ADHD 27%, control group 11%).

Some authors have argued that childhood ADHD is a developmental precursor of later antisocial behavior even in the absence of comorbid ODD or CD in childhood. For example, in a longitudinal study, Mannuzza et al. [8] demonstrated that in children with ADHD, even low to moderate levels of antisocial behaviors were associated with an increased risk for CD in adolescence. However, other studies found that a childhood diagnosis of ADHD is only predictive of delinquent behavior in adulthood if it is accompanied by early conduct behavior problems [9, 10]. Interestingly, early conduct problems can be considered as a general risk factor, since the predictive value of dissocial behavior for later delinquency has not only been reported in samples of children and adolescents with ADHD [11]. Low intelligence, negative parenting practices (e.g., poor parental supervision, punitive or erratic parental discipline, parental rejection and hostility), parental conflict, parental mental health problems, witnessing or being a victim of violence in childhood, peer delinquency, and a history of substance use have been identified as further risk factors for violence, offending, and delinquency [11–15].

An important question is whether ADHD treatment has a preventive effect on the development of delinquent behavior. A systematic review by Shaw et al. [16] investigated the impact of any ADHD treatment on long-term outcomes (defined as 2 years or more). Overall, individuals with treated ADHD experienced more favorable outcomes than those with untreated ADHD. However, treatment was only beneficial for outcomes relating to driving, obesity, self-esteem, social and academic functioning, and drug use/addictive behavior. Results regarding antisocial behavior (e.g., delinquency, self-reported crimes, arrests, and detainment) were inconsistent: only 50% of the studies that assessed antisocial behavior reported a beneficial effect of ADHD treatment on this outcome measure. Since the personal and societal costs of antisocial behavior are high, more research is needed to understand which factors influence the effectiveness of ADHD treatment in preventing delinquent behavior and to determine how children in need of specialized support can be identified.

The Multimodal Treatment Study of Children with ADHD (MTA study [17]) is one of the few studies to have followed children treated for ADHD into adulthood. Children with a diagnosis of ADHD (*n* = 579, aged 7–9 years) were randomly assigned to either medication management, intensive behavioral treatment, a combination of both, or standard community care. After a 14-month active treatment phase, the MTA study continued as a naturalistic follow-up study. In young adulthood, the MTA sample reported having had more police contacts and having been in jail more often than a local normative comparison group (police contact: ADHD 14% vs. LNCG 9%; jail time: ADHD 4% vs. LNCG 2%) but these group differences were not significant [18], suggesting that the treatment had a preventive effect. In addition, it was found that the likelihood of justice involvement in young adulthood was increased by low childhood household income and a conflicting parent–child relationship [19]. Interestingly, the effects of these two factors were comparable for the ADHD group and the LNCG group [19], suggesting that these risk factors were not specific for children with ADHD.

The present study focuses on the lifetime prevalence of delinquent behavior in children who participated in the Cologne Adaptive Multimodal Treatment Study (CAMT). In the CAMT Study, 75 children with ADHD (6–10 years of age) received individually tailored multimodal ADHD treatment consisting of medication management and/or behavioral interventions [20]. They were reassessed 18 months [21], 8 years [22] and 18 years [23] after the active treatment phase. At the 8-year follow-up, about one-third (35%) of the CAMT sample (aged between 15 and 22 years) reported having had one or more police contacts, and 16% reported having been convicted at least once. At the 18-year follow-up, the CAMT sample (now 22–32 years of age) showed substantial ADHD and ODD/CD symptom reduction. The majority (73%) did not qualify for a full DSM-5 [24] diagnosis of ADHD in adulthood, and the prevalence of ODD/CD was reduced from 61% at study entry to 13% at the 18-year FU. However, CAMT participants, as a group, had poor educational and occupational outcomes, and they had come into contact with the justice system more often than expected based on the adult outcomes of the MTA sample [18]. In young adulthood, 46% of the CAMT participants reported having had at least one police contact, and one-third of the sample (33%) reported having been charged or convicted for an offense, most often because of an assault with bodily harm, theft or drug use [23].

To summarize, a substantial proportion of CAMT participants had poor outcomes in adolescence and young adulthood, even though they had received individually tailored ADHD treatment in childhood. Therefore, we were interested to find out which factors predict delinquent behavior in the CAMT sample to improve the early identification of children in need of additional support. Based on self-reports of delinquent behavior at the 18-year follow-up, participants were categorized into two distinct groups: (1) no justice system involvement at any time (non-delinquent group, *n* = 34), and (2) justice system involvement (lifetime) reported at the 18-year follow-up (delinquent group, *n* = 25). Variables assessed in childhood after the active treatment phase (post-intervention) were used as possible predictor variables.

Based on previous studies showing that ADHD is only predictive of delinquent behavior if it is accompanied by early conduct behavior problems [9, 10], we hypothesized that CD-related behaviors in childhood, but not ADHD symptom severity, would predict later delinquency. In addition, a range of psychosocial variables found to be associated with delinquent behavior in previous studies [14] were considered as predictor variables. These include low intelligence, poor parental supervision/inconsistent parenting, lack of warmth in the parent–child relationship, parental mental illness, or partnership conflict. Data were analyzed in two steps. First, we determined which post-intervention measures were significantly associated with group membership (non-delinquent, delinquent). Next, we entered significant variables as possible predictor variables into a Chi-squared automatic interaction detector (CHAID) analysis, which is a data-mining technique that segments participants into homogenous subgroups.

## Methods

### Design of the CAMT Study

The Cologne Adaptive Multimodal Treatment (CAMT) Study used an adaptive research design with variable treatment intensity within a stepped care approach. A detailed description of the initial study is provided by Döpfner et al. [20]. The active treatment phase comprised four 6-week treatment phases. All families (*N* = 75) received six 50-min sessions of psychoeducation in the first treatment step. They were then partly randomized to either pharmacological treatment (*n* = 28) or six sessions of manualized behavior therapy (*n* = 45) in the next treatment step. The treatment in the following two steps was chosen based on the outcome of the previous steps. Semi-structured interviews and DSM-based symptom rating scales were used to assess treatment effectiveness after each treatment step. Depending on the outcomes, the weekly treatment could be terminated (full responder), new treatment components could be added (partial responder), or non-effective components could be omitted and new treatments added (non-responder). If the individually tailored behavior therapy was effective (full response), this treatment was continued (maximum 30 sessions). If behavior therapy was only partially effective (partial response), methylphenidate medication was added in the next treatment step. In the case of non-response to behavior therapy, a switch to pharmacological treatment was planned; however, there were no non-responders to behavior therapy. A similar procedure was conducted in children who started with medication management (medication treatment plus counselling on medication use and further psychoeducation). If medication was effective (full responder), participation in the active treatment phase was terminated and medication management was continued. If medication was partially effective (partial responder), behavior therapy was added in the next treatment steps. If medication was not effective (non-responder), medication was ceased and replaced by behavior therapy, because drugs other than methylphenidate were not licensed at the time of the investigation (in the 1990s). The adaptive approach with variable treatment intensity was continued after the active treatment phase, i.e., medication management and behavior therapy were continued by the same therapists and physicians but within the framework of routine care if indicated.

### Treatment conditions

The active treatment phase comprised six 50-min sessions of psychoeducation (PE, Step 1) followed by medication and PE (MED + PE) and/or behavior therapy (BT) in Steps 2–4. A comprehensive description can be found in Döpfner et al. [20]. In brief, PE aimed at providing information about ADHD, its individual causes and maintaining factors, building positive therapist–patient relationships, and identifying individual problem situations at home. In MED + PE, the effect of methylphenidate medication was assessed by single-blind placebo trials. Immediate-release methylphenidate (IR-MPH) was used because this was the only licensed medication for ADHD treatment in Germany when the CAMT Study started in 1990. If medication was effective during the morning, an afternoon dosage could be added. The daily dosage varied between 5 and 30 mg (mean daily dosage: 15 mg). In BT, components of a standardized treatment manual [25] were selected according to the individual needs of each child and family. The manual included cognitive behavior therapy for the child (e.g., self-instructional training), as well as family- and school-based interventions (e.g., implementing positive parent–child interactions, implementing rules and communicating commands effectively, compiling appropriate positive and negative consequences of child behavior, using token economies, response cost systems, time-out, and daily report cards).

### Participants and procedures

#### Initial study

Seventy-five children participated in the CAMT Study (93% boys, age range from 6 to 10 years, mean age = 8.3 years). All children had been referred to the outpatient unit of the Department of Child and Adolescent Psychiatry, University Hospital Cologne, Germany, for the treatment of inattentive, impulsive, and/or hyperactive behaviors. Inclusion criteria were: (a) age 6–10 years; (b) attending the first, second, third or fourth school grade; (c) a nonverbal IQ of 80 or higher; and (d) fulfillment of DSM-III-R [26] or ICD-10 [27] criteria for ADHD. ADHD symptoms were assessed through semi-structured interviews with parents and teachers (DISYPS [28]). All children met diagnostic criteria for a DSM-III-R diagnosis of ADHD according to either parent or teacher interview. In addition, 46 children (61%) fulfilled the criteria for a DSM-III-R diagnosis of ODD or CD, according to the parent interview.

#### 18-year follow-up [FU] study

The 18-year FU was conducted 13–24 years (*M* = 17.7 years, SD = 1.8) after the active treatment phase. The large range in follow-up time resulted from the fact that initial study recruitment and the 18-year follow-up study each spread over several years. Sixty-nine participants (92% of 75), who were aged 22–32 years at the 18-year FU, provided information on number of police contacts and convictions (lifetime prevalence) in a structured telephone interview. Nine participants (13.0%) reported having had one or more police contacts (without conviction) and 23 participants (33.3%) reported having been convicted at least once (7.2% with imprisonment, 26.1% without imprisonment). Among those who had been in contact with the police (*n* = 32), the most common offenses were assault with bodily harm (*n* = 15, 47%), drug use (*n* = 13, 41%), and theft (*n* = 13, 41%). Döpfner et al. [23] provide a detailed description of the results, including educational, occupational and health-related outcomes.

#### Current analysis

Fifty-nine participants had complete datasets for the variables of interest and were included in the analysis reported in this article. Of these, 54 (91.5%) were male and 5 (8.5%) were female.

### Group allocation

For the current analysis, participants were allocated into two groups based on self-reports of police contacts and convictions at the 18-year follow-up. The first group comprises participants without justice system involvement (e.g., no police contacts, no charges or convictions) at any time (non-delinquent group, *n* = 34, 57.6%). The second group comprises participants with justice system involvement (e.g., at least one police contact or conviction) at the 18-year follow-up (delinquent group, *n* = 25, 42.4%).

### Predictor variables

All variables considered as potential predictor variables were assessed at ages 6–10 years immediately after the active treatment phase (post-intervention).

#### Intelligence

Intelligence was assessed with the German version of the Kaufman Assessment Battery for Children (K-ABC [29]). The K-ABC is composed of two summative scales: the Mental Processing Composite (MPC), comprising Sequential and Simultaneous Processing subscales, which provides a global measure of the child’s cognitive ability, and the Achievement scale, an assessment of knowledge of facts, language concepts, and school-related skills. In addition, a Nonverbal subscale is available, which is limited to subtests that require gestural responses. All standard scores have a standardized mean of *M* = 100 and standard deviation of SD = 15.

#### ADHD rating scale (FBB-ADHS)

The parent and teacher rating scale FBB-ADHS is part of the German ICD- and DSM-based Diagnostic System for the Assessment of Mental Disorders in Children and Adolescents (DISYPS [28]). The first version of the FBB-ADHS, which was available when the active treatment phase was performed, contained 23 items [30]. The items assess the occurrence of ADHD symptoms (e.g., “often blurts out answers to questions”) and are rated on 4-point Likert scales ranging from 0 (*not at all*) to 3 (*very much*). Item scores are averaged to yield a scale score that varies from 0 to 3. Research has shown that the FBB-ADHS is a reliable (internally consistent) and valid instrument [30, 31].

#### ODD/CD rating scale (FBB-SSV)

The parent and teacher rating scale FBB-SSV is also part of the German ICD- and DSM-based Diagnostic System for the Assessment of Mental Disorders in Children and Adolescents [28]. The first version of the FBB-SSV, which was available when the active treatment phase was performed, comprised 24 items [30]. Ten items assess symptoms of ODD (e.g., “often argues with adults”) and 14 items assess symptoms of CD (e.g., “has broken into someone else’s house, building, or car”). All items are rated on 4-point Likert scales ranging from 0 (*not at all*) to 3 (*very much*). Item scores are averaged to yield a scale score that varies from 0 to 3. The slightly revised FBB-SSV [28] has good reliability and validity [32, 33].

#### Home Situations Questionnaire (HSQ)

The Home Situations Questionnaire (HSQ [34], German version [34]) was used to assess the presence and intensity of behavioral non-compliance in everyday settings. The HSQ consists of 16 items describing different situations or settings that are common in the home environment (e.g., getting dressed). Parents are asked to indicate whether the child has problems with compliance in these situations and, if so, to rate the severity on a 9-point Likert scale (1–9), with higher scores indicating greater non-compliance. Reliability and validity were demonstrated in clinical and field samples [35, 36].

#### Homework Problem Checklist (HPC)

The parent-rated HPC [37] assesses problem behavior during homework. The checklist lists 20 statements about problems that may occur when a student does homework (e.g., “Fails to bring homework assignment and necessary materials”, “daydreams or plays with objects”). The parent is asked to rate the frequency of occurrence of each problem using a 4-point rating scale: 0 (*never*), 1 (*at times*), 2 (*often*), and 3 (*very often*). Reliability and validity of the German version were demonstrated in clinical and field samples [1].

#### Child Behavior Checklist (CBCL)

The CBCL 4–18 [38] (German: [39, 40]) consists of 120 items that describe typical behavioral and emotional problems. Parents rate each item as 0 (*not true*), 1 (s*omewhat or sometimes true*), or 2 (*very true or often true*). There are two broad-band syndrome scales (Externalizing and Internalizing Problems) and eight syndrome scales (Withdrawn, Somatic Complaints, Anxious/Depressed, Social Problems, Thought Problems, Attention Problems, Delinquent Behavior, and Aggressive Behavior). Higher scores indicate greater problems. The German version of the CBCL 4–18 has good reliability and validity [40].

#### Teacher Report Form (TRF)

The TRF [41] (German: [40, 42]) measures teacher-reported behavioral and emotional problems. The 118 items are rated as 0 (not true), 1 (somewhat or sometimes true), or 2 (very true or often true). Analogous to the CBCL, the TRF yields two broad-band syndromes (Externalizing and Internalizing Problems) and eight syndrome scales (Withdrawn, Somatic Complaints, Anxious/Depressed, Social Problems, Thought Problems, Attention Problems, Delinquent Behavior, and Aggressive Behavior). Higher scores indicate greater problems. The German version of the TRF school-age form has good reliability and validity [40].

#### Clinical ratings

After the active treatment phase, the therapist who had been working with the family during the active treatment phase conducted a parent interview. The interview included an 8-item evaluation of externalizing child behavior at home and at school (e.g., “Aggressive behavior towards teachers”, “Aggressive behavior towards peers”), a 7-item evaluation of adverse family conditions (e.g., “Lack of warmth in the parent–child relationship”, “Poor parental supervision and inconsistent parenting”, “Insufficient learning opportunities and experiences”), and a 5-item evaluation of adverse school conditions (e.g., “Hostility towards and scapegoating of child by teacher or peers”, “Poor teacher–child-relationship”). All items were rated on a 5-point scale ranging from 0 (not at all) to 4 (very much). Single items were not summed to scale scores since we were interested to find out which aspects of child, family and school functioning are related to later delinquency.

In addition, the therapist evaluated the prognosis for the child’s overall development on a 5-point scale. A score of 1 was given for a very positive prognosis in multiple contexts (family, school, peers), and a score of 5 was given if substantial impairments in multiple domains of functioning were predicted that would require persistent support or mentoring. The therapist also indicated the treatment duration (operationalized as the number of treatment phases during the active treatment), whether the participant was prescribed stimulant medication (yes/no) and whether the family had discontinued the treatment (yes/no).

#### Symptom count diagnoses

Based on the parent and teacher rating scales FBB-ADHS and FBB-SSV (described above), it was determined whether children met the symptom count diagnostic criteria for hyperkinetic conduct disorder (ICD-10: F90.1) or ODD/CD according to ICD-10 and DSM-III-R (ICD-10: F91.x, DSM-III-R: 312). ICD-10 and DSM-II-R were the current editions of the diagnostic manuals when we conducted the post-intervention assessment.

### Data analyses

Fifty-nine participants (78.7% of 75) had complete datasets for the variables of interest and were included in the present analyses. We first determined associations between group membership (delinquent/non-delinquent) and possible predictor variables. The phi coefficient (*φ*) is a measure of association for two binary variables and was used for dichotomous variables (e.g., Stimulant medication during active treatment phase: yes/no). The eta coefficient (*ɳ*) was used to determine whether a relationship exists between group membership and measures on an interval scale (e.g., rating scales). Spearman’s rank correlation coefficient (*r*_s_) was used as a measure of association between group membership and non-normally distributed variables (e.g., number of treatment steps during active treatment phase). For most variables (e.g., intelligence, parent ratings, and teacher ratings), scale scores were analyzed. Clinical ratings were analyzed on a single-item basis. Child gender was not included in the analyses because the present sample included only 5 girls, as opposed to 54 boys. All tests used a two-sided significance level. We did not apply a correction for multiple testing (e.g., Bonferroni) as we aimed to explore these data to identify variables for further analyses.

Variables that correlated significantly with group membership were then entered as possible predictor variables in a Chi-squared automatic interaction detector (CHAID) analysis. CHAID analysis is a decision tree technique that can be used to determine whether the independent variables predict the dependent or criterion variable, in this case group membership. CHAID divides a population into mutually exclusive and exhaustive subpopulations (nodes) based on categories of the best predictor and then splits each of these subpopulations into smaller groups based on further predictors, generating a visual decision tree [43]. First, a Chi-squared test is used to determine the best predictor (i.e., the one with the most significant *p* value). The sample is then partitioned into two or more nodes based on the categories of the predictor variable. Subsets that are not significantly different are combined. This procedure is repeated for each node in a stepwise manner until a node can no longer be split into significantly different subsets. These terminal nodes represent the best classification predictions. Due to the relatively small sample size (*N* = 59), tree depth was limited to three levels. The minimum number of cases in the parent node was set to six, and the minimum number of cases in the child node to two. A *p* value < 0.10 in the Chi-squared statistic, adopting Bonferroni’s correction, was considered significant for node-splitting purposes.

CHAID analysis was chosen as the method for data analyses, because this method partitions a sample into mutually exclusive subgroups based on a categorical or ordinal outcome and several categorical or ordinal predictor variables. Unlike regression analysis, CHAID analysis is non-parametric and does not require the data to be normally distributed. In addition, CHAID generates a visual decision tree that is easy to interpret and provides cutoff values to discriminate between subgroups. All statistical procedures were performed using IBM-SPSS Statistics 23.0. For all variables, raw scores were used.

## Results

### Description of delinquent behavior

At the 18-year FU, only male participants (*n* = 25, 42.4%) reported having had at least one police contact and/or conviction. Details on the type of delinquent behavior in which they engaged and/or the offenses committed were provided by 23 participants (39.0%). The most common types of offense were drug-related offenses (*n* = 10, 16.9%) and assaults with bodily harm (*n* = 10, 16.9%) followed by offenses related to driving (*n* = 6, 10.2%), theft (*n* = 6, 10.2%), damage of property (*n* = 3, 5.1%), fraud (*n* = 3, 5.1%), burglary (*n* = 2, 3.4%), violation of the German Weapons Act (*n* = 1, 1.7%), and trespassing (*n* = 3, 5.1%). As 12 participants (20.3%) provided multiple answers, the total exceeds 100%. Four participants (6.8%) had been imprisoned.

### Correlation analyses

Table 1 shows the results of the correlation analyses. Compared to the delinquent group, the non-delinquent group had significantly higher scores on the Achievement scale (Eta = 0.30, *p* = 0.021) and the Nonverbal scale (Eta = 0.29, *p* = 0.028) of the K-ABC. The non-delinquent group also had significantly lower scores on the CBCL Delinquent behavior scale (Eta = 0.36, *p* > 0.001), the TRF Delinquent behavior scale (Eta = 0.30, *p* = 0.037), and the TRF Externalizing scale (Eta = 0.26, *p* = 0.043), as well as on the clinician-rated items “Aggressive behavior towards teachers” (*r*_s_ = 0.30, *p* = 0.022), “Aggressive behavior towards peers” (*r*_s_ = 0.30, *p* = 0.019), “Mental disorder of other family member” (*r*_s_ = 0.27, *p* = 0.044), “Lack of warmth in the parent–child relationship” (*r*_s_ = 0.33, *p* = 0.010), “Poor parental supervision and inconsistent parenting practices” (*r*_s_ = 0.28, *p* = 0.034), and “Insufficient learning opportunities and experiences” (*r*_s_ = 0.27, *p* = 0.030). In addition, significantly fewer participants in the non-delinquent group qualified for a symptom count diagnosis of ADHD + ODD according to ICD-10 criteria (F90.1, parent rating, Phi = 0.34, *p* = 0.013), a symptom count diagnosis of ODD/CD according to ICD-10 criteria (F91.x, teacher rating, Phi = 0.36, *p* = 0.006), or a symptom count diagnosis of CD according to DSM-III-R criteria (312, parent rating: Phi = 0.38, *p* = 0.003, teacher rating: Phi = 0.32, *p* = 0.013) compared to the delinquent group.

**Table 1:** Correlations between potential predictor variables assessed in childhood (post-intervention) and group membership (non-delinquent/delinquent)

Post-intervention variables	Non-delinquent group (*n* = 34)		Delinquent group (*n* = 25)		Correlation coefficient^a^
Age in years	*M* = 9.7	SD = 1.0	*M* = 9.4	SD = 1.2	Eta = 0.14, p = 0.279
Intelligence (K-ABC)					
Mental processing composite (MPC)	*M* = 104.4	SD = 7.4	*M* = 99.9	SD = 11.2	Eta = 0.24, p = 0.071
Achievement scale	*M* = 104.2	SD = 10.1	*M* = 96.3	SD = 15.5	Eta = 0.30, p = 0.021
Nonverbal subscale	*M* = 106.5	SD = 9.8	*M* = 99.7	SD = 13.0	Eta = 0.29, p = 0.028
Treatment during active treatment phase					
Received stimulant medication	*n* = 17	50.0%	*n* = 17	68.0%	Phi = 0.18, p = 0.167
Treatment duration (no. of treatment steps)	*M* = 4.4	SD = 0.9	*M* = 4.8	SD = 0.8	*r*_s_ = 0.25, p = 0.065
Discontinuation of treatment	*n* = 2	6.9%	*n* = 3	15.8%	Phi = 0.16, p = 0.292
Parent ratings					
Home Situations Questionnaire (HSQ)	*M* = 2.1	SD = 1.2	*M* = 2.8	SD = 1.8	Eta = 0.23, p = 0.100
Homework Problem Checklist (HPC)	*M* = 1.1	SD = 0.5	*M* = 1.2	SD = 0.6	Eta = 0.15, p = 0.257
ADHD (FBB-ADHS)	*M* = 1.3	SD = 0.4	*M* = 1.5	SD = 0.5	Eta = 0.25, p = 0.053
ODD/CD (FBB-SSV)	*M* = 0.6	SD = 0.3	*M* = 0.8	SD = 0.5	Eta = 0.17, p = 0.237
CBCL attention problems	*M* = 6.3	SD = 2.8	*M* = 6.8	SD = 4.0	Eta = 0.07, p = 0.592
CBCL aggressive behavior	*M* = 14.0	SD = 7.2	*M* = 15.5	SD = 9.6	Eta = 0.09, p = 0.481
CBCL delinquent behavior	*M* = 2.7	SD = 2.2	*M* = 4.9	SD = 3.4	Eta = 0.36, p > 0.001
CBCL internalizing	*M* = 8.1	SD = 7.7	*M* = 6.6	SD = 6.5	Eta = 0.11, p = 0.418
CBCL externalizing	*M* = 16.7	SD = 8.9	*M* = 20.4	SD = 12.5	Eta = 0.18, p = 0.185
Teacher ratings					
ADHD (FBB-ADHS)	*M* = 1.2	SD = 0.5	*M* = 1.1	SD = 0.6	Eta = 0.12, p = 0.383
ODD/CD (FBB-SSV)	*M* = 0.4	SD = 0.3	*M* = 0.5	SD = 0.4	Eta = 0.13, p = 0.342
TRF attention problems	*M* = 12.4	SD = 7.2	*M* = 15.4	SD = 8.1	Eta = 0.20, p = 0.138
TRF aggressive behavior	*M* = 11.2	SD = 8.6	*M* = 15.8	SD = 10.2	Eta = 0.24, p = 0.065
TRF delinquent behavior	*M* = 1.5	SD = 1.4	*M* = 2.8	SD = 2.7	Eta = 0.30, p = 0.037
TRF internalizing	*M* = 7.6	SD = 6.0	*M* = 7.8	SD = 6.2	Eta = 0.01, p = 0.915
TRF externalizing	*M* = 12.8	SD = 9.5	*M* = 18.6	SD = 12.4	Eta = 0.26, p = 0.043
Clinical ratings of child behavior (8 items)					
Poor attention, hyperactivity and/or impulsivity at home	*M*_rank_ = 29.18		*M*_rank_ = 31.12		*r*_s_ = 0.06, p = 0.642
Oppositional and/or defiant behavior towards parents	*M*_rank_ = 27.79		*M*_rank_ = 33.00		*r*_s_ = 0.16, p = 0.230
Aggressive behavior towards parents	*M*_rank_ = 28.16		*M*_rank_ = 32.50		*r*_s_ = 0.14, p = 0.280
Aggressive behavior towards other family members	*M*_rank_ = 27.53		*M*_rank_ = 33.36		*r*_s_ = 0.19, p = 0.153
Poor attention, hyperactivity and/or impulsivity at school	*M*_rank_ = 27.38		*M*_rank_ = 32.30		*r*_s_ = 0.15, p = 0.249
Oppositional and/or defiant behavior during class time	*M*_rank_ = 27.74		*M*_rank_ = 33.08		*r*_s_ = 0.17, p = 0.199
Aggressive behavior towards teachers	*M*_rank_ = 27.06		*M*_rank_ = 34.00		*r*_s_ = 0.30, p = 0.022
Aggressive behavior towards peers	*M*_rank_ = 25.85		*M*_rank_ = 35.64		*r*_s_ = 0.30, p = 0.019
Clinical evaluation of family conditions (7 items)					
Parental mental disorder	*M*_rank_ = 31.50		*M*_rank_ = 27.96		*r*_s_ = − 0.12, p = 0.376
Mental disorder of other family member	*M*_rank_ = 27.18		*M*_rank_ = 32.56		*r*_s_ = 0.27, p = 0.044
Poor parent relationship	*M*_rank_ = 29.99		*M*_rank_ = 30.02		*r*_s_ = 0.00, p = 0.994
Lack of warmth in the parent–child relationship	*M*_rank_ = 25.49		*M*_rank_ = 34.14		*r*_s_ = 0.33, p = 0.010
Parental overprotection	*M*_rank_ = 30.68		*M*_rank_ = 29.08		*r*_s_ = − 0.07, p = 0.620
Poor parental supervision and inconsistent parenting practices	*M*_rank_ = 25.96		*M*_rank_ = 34.52		*r*_s_ = 0.28, p = 0.034
Insufficient learning opportunities and experiences	*M*_rank_ = 27.07		*M*_rank_ = 33.98		*r*_s_ = 0.27, p = 0.030
Clinical evaluation of school conditions (5 items)					
Lack of warmth in the teacher–child relationship	*M*_rank_ = 30.22		*M*_rank_ = 29.70		*r*_s_ = − 0.03, p = 0.849
Inadequate supervision/control and inconsistent practices	*M*_rank_ = 30.21		*M*_rank_ = 29.72		*r*_s_ = − 0.04, p = 0.781
Intellectual overload of child	*M*_rank_ = 28.32		*M*_rank_ = 32.28		*r*_s_ = 0.24, p = 0.070
Hostility towards and scapegoating of child by teacher or peers	*M*_rank_ = 28.3		*M*_rank_ = 32.5		*r*_s_ = 0.23, p = 0.085
Poor teacher–parent relationship	*M*_rank_ = 27.74		*M*_rank_ = 33.08		*r*_s_ = 0.21, p = 0.113
Clinical prognosis for the child’s overall development	*M*_rank_ = 22.0		*M*_rank_ = 28.3		*r*_s_ = 0.27, p = 0.055
Symptom count diagnoses					
Hyperkinetic CD (ICD-10: F90.1, parent rating)	*n* = 1	3.2%	*n* = 6	26.1%	Phi = 0.34, p = 0.013
Hyperkinetic CD (ICD-10: F90.1, teacher rating)	*n* = 2	6.3%	*n* = 3	12.0%	Phi = 0.10, p = 0.446
ODD/CD (ICD-10: F91.x, parent rating)	*n* = 4	11.8%	*n* = 8	32.0%	Phi = 0.25, p = 0.056
ODD/CD (ICD-10: F91.x, teacher rating)	*n* = 0	0.0%	*n* = 5	20.0%	Phi = 0.36, p = 0.006
CD (DSM-III-R: 312, parent rating)	*n* = 2	5.9%	*n* = 9	36.0%	Phi = 0.38, p = 0.003
CD (DSM-III-R: 312, teacher rating)	*n* = 1	2.9%	*n* = 6	24.0%	Phi = 0.32, p = 0.013

*K-ABC* Kaufman Assessment Battery for Children, *ADHD* Attention-Deficit/Hyperactivity Disorder, *ODD* Oppositional Defiant Disorder, *CD* Conduct Disorder, *CBCL* Child Behavior Checklist, *TRF* Teacher Report Form

^a^Depending on the outcome measure, Spearman’s rank correlation coefficient (*r*_s_), the phi coefficient (*φ*) or the eta coefficient (*ɳ*) were determined

### CHAID analysis

**Fig. 1:**
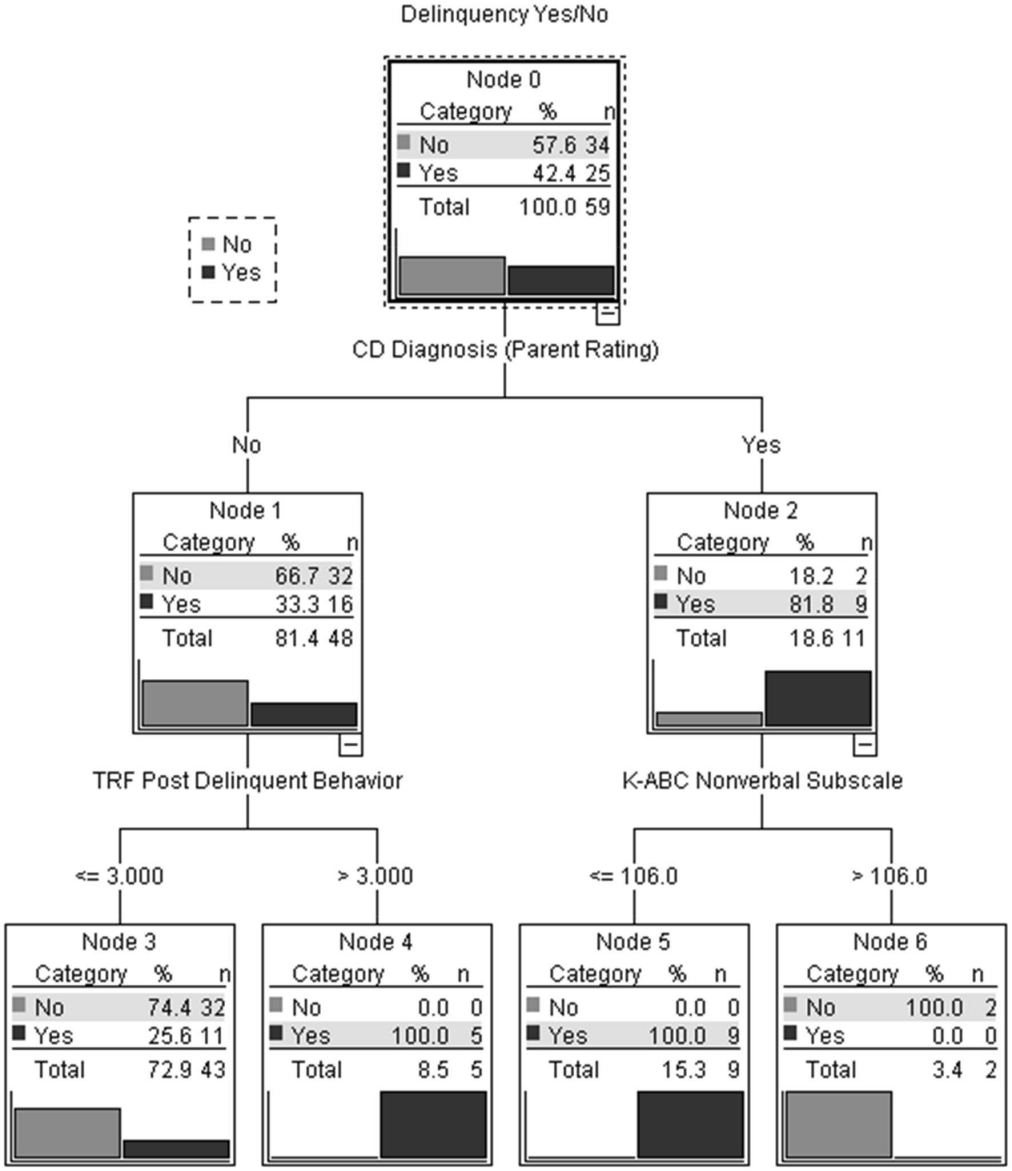
CHAID decision tree for group membership (non-delinquent/delinquent) (*n* = 59)

Figure 1 shows the CHAID decision tree for the prediction of group membership. The best predictor (i.e., the most significant variable) is the presence of a symptom count diagnosis of CD according to DSM-III-R criteria based on parent ratings at post-intervention (Chi^2^ = 8.62, *p* < 0.001). For participants not meeting the symptom count diagnostic criteria for CD according to parent ratings (node 1), scores on the TRF Delinquent behavior scale were detected as a significant predictor variable (Chi^2^ = 11.16, *p* < 0.001). Participants without a diagnosis of CD and with a low score (≤ 3.0) on the TRF Delinquent behavior scale tended to become non-delinquent (node 3 [terminal node]: *n* = 32 [74%] non-delinquent). Participants without a CD diagnosis but with a high score (> 3.0) on the TRF Delinquent behavior scale reported delinquent behavior at the 18-year FU (node 4 [terminal node]: *n* = 5 [100%] delinquent).

For participants meeting the symptom count diagnostic criteria for CD according to DSM-III-R criteria based on parent ratings at post-intervention (node 2), nonverbal intelligence (K-ABC Nonverbal subscale) was detected as a significant predictor variable (Chi^2^ = 11.00, *p* < 0.001). Participants with a diagnosis of CD and a nonverbal intelligence scale score of ≤ 106 reported delinquent behavior (node 5 [terminal node]: *n* = 9 [100%] delinquent), while participants with a diagnosis of CD and a nonverbal intelligence scale score of > 106 did not (node 6 [terminal node]: *n* = 2 [100%] non-delinquent).

When the predictor variables and cutoffs specified in the CHAID analysis were used to predict group membership, 81.4% of cases were classified correctly. All cases (100%) of the non-delinquent group were classified correctly (specificity = 1). Of the delinquent group, *n* = 14 (56%) were correctly classified, while *n* = 11 (44%) were incorrectly classified by the tree (sensitivity = 0.56). This corresponds to a positive predictive value of 1 and a negative predictive value of 0.76.

## Discussion

ADHD is associated with increased rates of oppositional, aggressive, and rule-breaking behavior problems in childhood [4] and, possibly due to this association, a higher risk of being arrested, convicted, or incarcerated during adolescence or adulthood [5, 6]. There is some evidence that ADHD treatment has a preventive effect on the development of delinquent behavior. For example, Hechtman et al. [18] found that the MTA sample did not report more police contacts in adulthood than a local normative comparison group. However, a systematic review by Shaw et al. [16] revealed mixed findings, with 50% of studies failing to find a beneficial effect of ADHD treatment on antisocial behavior. Since the personal and societal costs of dissocial behavior are high, the present study aimed to identify factors that predict delinquent behavior in young adults who had been diagnosed and treated for ADHD in childhood. The participants (*N* = 59) had received individualized ADHD treatment within the Cologne Adaptive Multimodal Treatment Study (CAMT) during childhood and were reassessed in adulthood (18-year FU). As we reported previously [23], the CAMT sample had poorer educational and occupational outcomes in adulthood and more criminal justice system involvement than expected (e.g., lifetime convictions: 33%), despite symptomatic improvement.

In the present study, we first determined which childhood measures (assessed at post-intervention) were significantly associated with lifetime delinquency reported in adulthood (delinquent group: *n* = 25, non-delinquent group: *n* = 34). Compared to the delinquent group, the non-delinquent group had significantly higher scores on two measures of intelligence (Achievement scale and Nonverbal scale of the K-ABC). The delinquent group had significantly higher scores on parent and teacher ratings of delinquent and externalizing behavior problems, and on clinicians’ ratings of aggressive behavior at school, mental disorders of family members, lack of warmth in the parent–child relationship, poor parental supervision/inconsistent parenting practices, and insufficient learning opportunities. In addition, participants in the delinquent group qualified significantly more often for a symptom count diagnosis of hyperkinetic conduct disorder (ICD-10: F90.1) based on parent ratings, a symptom count diagnosis of ODD/CD (ICD-10: F91.x) based on teacher ratings, and a symptom count diagnosis of CD (DSM-III-R: 312) based on parent and/or teacher ratings. These results are consistent with previous findings on general risk factors for violence, offending, and delinquency [11–15]. Importantly, ADHD symptom severity in childhood was not significantly associated with later delinquency.

We then entered childhood variables that were significantly associated with group membership as possible predictor variables into a Chi-squared automatic interaction detector (CHAID) analysis. As we hypothesized based on previous studies [9, 10], early conduct problems were found to be a significant predictor of later delinquency. More specifically, lifetime reports of police contacts and convictions in adulthood were best predicted by (a) meeting the symptom count diagnostic criteria for conduct disorder (CD) according to parent ratings in combination with a nonverbal intelligence of IQ ≤ 106 at post-intervention, and (b) delinquent behavior problems according to teacher ratings at post-intervention. When these predictor variables and cutoffs were used to predict group membership, 81% of cases were classified correctly.

To summarize, we found that early conduct problems reported by parents or teachers after completion of ADHD treatment, but not ADHD symptom severity, were significant predictors of later delinquency or non-delinquency. Consistent with previous studies [14], low nonverbal intelligence was found to be an additional risk factor. These findings support the hypothesis that a childhood diagnosis of ADHD is only predictive of delinquent behavior if it is accompanied by early conduct behavior problems, and hence that there is no direct association between ADHD and criminality [9]. In addition, our findings suggest that children with ADHD and comorbid conduct problems are in need of continued treatment with a focus on reducing dissocial behaviors. Psychological treatments such as social skills trainings and behavioral parent trainings that aim at decreasing harsh discipline and improving child-directed play skills, positive parenting, effective limit setting, and proactive control have been found to effectively reduce conduct problems in children and adolescents with CD [44]. In addition, pharmacological treatment with psychostimulants (e.g., methylphenidate) or atypical antipsychotics (e.g., risperidone) shows effects on conduct problems in children with ADHD and comorbid CD [45] and may be considered as a treatment option for children with severe forms of aggression.

It is important to note that our results regarding parenting and other family factors were partly unexpected. Previous studies showed that family factors such as living with someone with a mental health problem, and parenting styles such as poor parental supervision, cold parental attitude, and parental conflict, predict delinquency [14, 15]. The present study confirms these findings by showing that more than half of our childhood measures of family factors (i.e., mental disorder of a family member other than a parent, lack of warmth in the parent–child relationship, poor parental supervision/inconsistent parenting, and insufficient learning opportunities at home) were significantly associated with later delinquency. Yet, other family factors such as mental disorder of a parent and parental conflict were not associated with the development of delinquent behavior, and none of the family factors turned out to be among the best predictors in the CHAID analysis after early conduct behaviors were included in the CHAID model. The latter finding might be due to correlations between family factors and measures of conduct behavior. Another possible explanation is that we analyzed clinical ratings of family conditions on a single items basis. Research has shown that the multi-items scales often perform better in terms of predictive validity than single items [46]. The use of single items might also explain the lack of significant correlations between delinquent behavior in adulthood and clinical ratings of childhood behavior and school conditions.

In addition, none of the characteristics of the treatment provided in childhood (e.g., duration of treatment, treatment with stimulant medication as part of the multimodal treatment approach) were significantly associated with adult reports of delinquent behavior, possibly because all CAMT participants received treatment and there was not sufficient variance in the data to allow detection of long-term predictive effects. At end of the active treatment phase, both children who were treated with behavioral interventions only (PE + BT) and children who were treated with combined treatment (MED + PE or MED + PE + BT) showed significant reductions in parent and teacher-rated ODD/CD symptoms (effect sizes *d* = 0.3–1.0) [20]. Consequently, the results of the CAMT Study demonstrate that multimodal treatment has positive short-term effects on oppositional and rule-breaking behaviors in children with ADHD. Our results do not allow us to conclude whether certain aspects of the multimodal ADHD treatment had a preventive effect on delinquency later in life.

Previous studies demonstrated that behavioral programs that focus on parenting skills training, behavioral modeling, or behavioral contracting are effective in preventing persistent juvenile delinquency (effect sizes: *d* = 0.57–0.61 [47]). We, therefore, suggest that treatment for children with ADHD and comorbid conduct problems should include behavioral interventions that focus on reducing externalizing behavior problems. In the CAMT Study, all participants had received at least six sessions of psychoeducation, and most of them were then allocated to either manualized behavior therapy or medication management (medication treatment plus counseling on medication use and further psychoeducation) in the next treatment step. This design does not allow us to investigate whether behavioral interventions had a long-term preventive effect. It would be desirable for future studies to disentangle the preventive effects of medication and behavioral interventions in children with ADHD and other externalizing behavior problems. Further limitations of our study include the relatively small sample size, which might have limited the power to detect small effects and, therefore, the interpretation of our results.

Another limitation worth mentioning is the heterogeneity of delinquent behaviors and offenses reported by the delinquent group (e.g., drug-related offenses, assaults with bodily harm, offenses related to driving, theft, damage of property, and fraud). Due to the small size of subgroups (*n* ≤ 10) and the fact that 20% of participants reported multiple offenses, a subgroup analysis would not have been properly powered. Future research is needed to determine shared and unique predictors of different types of delinquent behaviors.

Finally, it is important to highlight that our sample was referred and treated for ADHD and, therefore, our results cannot be generalized to non-referred samples. Clinically referred samples often present more severe symptoms, are more impaired, and are more likely to have comorbid disorders than untreated samples [48, 49]. As expected, the prevalence of ODD/CD in the CAMT sample (61% at study entry) was higher than the rate of ODD/CD in community-based samples of individuals with ADHD [50]. It was, however, comparable to the rate of ODD/CD in the MTA sample (54%) [51], suggesting that the present sample is representative of clinically referred children with ADHD. We, therefore, conclude that our study adds valuable information about the prediction of delinquent behavior in clinically referred children with ADHD, which might help to improve treatment options. Since early conduct problems were found to be the best predictor of later delinquency, interventions that focus on promoting compliance and reducing oppositional and aggressive behaviors (e.g., parent management training, cognitive behavioral interventions, social skills training) should be an essential component of the treatment provided to children with ADHD and comorbid conduct problems.
